# Microdamage Repair and Remodeling Requires Mechanical Loading

**DOI:** 10.1359/jbmr.091016

**Published:** 2009-10-12

**Authors:** Erik I Waldorff, Katya B Christenson, Laura A Cooney, Steven A Goldstein

**Affiliations:** 1 Orthopaedic Research Laboratories, Department of Orthopaedic Surgery, University of MichiganAnn Arbor, MI, USA; 2 Internal Medicine, Division of Rheumatology, University of MichiganAnn Arbor, MI, USA

**Keywords:** microdamage, hindlimb suspension, targeted remodeling, RAT, stress fracture

## Abstract

Bone remodeling is necessary to avoid microdamage accumulation, which could lead to whole-bone failure. Previous studies have shown that this bone-repair mechanism is triggered by osteocyte apoptosis. Through the use of a rodent hindlimb suspension model and tibial four-point bending model, the effects of disuse on microdamage remodeling was examined. At day 0, male rats were assigned to one of three groups: weight bearing (WB), hindlimb suspension (HS), or hindlimb suspension with daily intermittent weight bearing following damage-inducing loading (HW). Within each group, the rats were further divided into subgroups corresponding to three sacrifice time points [day 14 (WB and HS only), day 18, or day 35]. At day 14, animals were anesthetized, and their left tibiae underwent cyclic four-point bending to produce fatigue-induced microdamage. At sacrifice, the tibiae were examined using 3D micro-computed tomography (µCT), flow cytometry, and histologic and immunohistochemical stains. The results indicate that only the WB and HW groups had a significant increase in intracortical TRAP-positive resorption pits following damage induction, which was paralleled by a significant decrease in microdamage over time in combination with a shift in the osteoclast lineage owing to a decrease in monocytes. These results demonstrate that osteocyte apoptosis may be insufficient for repair of microdamage without the stimulation provided through physiologic loading. In addition, this potentially could have clinical implications for the current therapeutic paradigm for treating stress fractures, where extended non-weight bearing is employed. © 2010 American Society for Bone and Mineral Research.

## Introduction

Osteoporotic fracture is a common and expensive health care problem, with 1.5 million fractures in the United States per year, with a predicted cost of $60 billion annually in the United States by 2025.(1) Yet the factors responsible for susceptibility to fracture remain incompletely understood.

The existence of microdamage within bone has been reported to induce localized osteocyte apoptosis surrounding individual microcracks,(2,3) which subsequently leads to targeted remodeling.(2,4–9) It has been proposed that whole-bone failure in osteoporosis may be a result of positive feedback between microdamage and the resulting remodeling that attempts to repair the damage.(10) Microdamage results in a loss of mechanical integrity of the bone tissue, followed by a potentially greater loss in continuum-level bone strength and/or stiffness owing to resorption at the beginning of the remodeling cycle. The reduced stiffness and strength may result in further damage or overt failure at lower loads than those required in the original intact bone, resulting in a positive-feedback process. This potentially could have important clinical implications. The relationship between microdamage accumulation and resorption may explain a portion of the increase in fracture risk in the elderly population. Hence whole-bone fracture risk potentially may increase if remodeling is altered. Aside from the alterations in remodeling activity owing to age,(11) bone loss associated with aging also may result from disuse owing to reductions in physical activity or infirmity.

Disuse models such as prolonged bed rest have shown that urinary levels of bone-formation markers decreased, whereas resorption markers and resistance to insulin-like growth factor 1 (IGF-1) increased, leading to cortical and cancellous bone loss.(12,13) Similar effects have been found in astronauts during long-term exposure to microgravity, during which disuse of the weight-bearing limbs is prevalent.(14,15) To simulate the disuse condition of bed rest and weightlessness, several groups have developed rodent hindlimb suspension models(16–18) that induce similar effects, such as increased resorption and decreased formation,(19) increased resistance to IGF-1,(10,20,21) and significant reduction of blood flow.(22) Disuse hindlimb suspension models also have been shown to decrease interstitial fluid flow owing to decreased pressure gradients.(23) Several studies suggest that convective transport by means of load-induced fluid flow may be necessary to provide sufficient transport of larger molecules such as proteins to and from osteocytes.(24,25) The decrease in interstitial fluid flow possibly could contribute to a lack of osteoclast activation during hindlimb suspension. It therefore was hypothesized that the removal of functional load would reduce or inhibit targeted remodeling.

Previous studies have shown that supine weight-bearing exercise within a lower body negative chamber (LBNP) counteracts bone loss associated with long-term bed rest.(26,27) However, daily standing for 1 or 2 hours per day during 28 days of hindlimb suspension does not alter the deterioration of cortical bone.(28) Early clinical evidence for recovery of bone repair in a disuse setting with moderate physiologic loading comes from the treatment of running-related stress fractures. Previous treatment methods for stress fractures associated with long-distance running prescribed up to 12 weeks of therapy (dominantly non–weight bearing) before returning to a normal running schedule.(29) However, a recent study (without an experimental basis) decreased the recovery period by implementing earlier cross-training and enabled the athlete to return to function in only 7 weeks.(30) It therefore also was hypothesized that moderate physiologic loading could rescue the potentially impaired microdamage repair process during disuse.

The purpose of this study thus was to examine the effects of disuse and intermittent weight bearing on bone remodeling in response to microdamage, potentially providing clinically important insight into the relationship between microdamage accumulation and increased fracture risk in states of disuse.

## Materials and Methods

### Animals

Male 6-month-old adult Sprague-Dawley rats (350 to 450 g) were obtained from Harlan Laboratories (Somerville, NJ, USA). Animals were allowed to acclimate to our animal facility for at least 3 days before being included in the experiment. The procedures used in this study were approved by the University Committee on Use and Care of Animals at the University of Michigan. Animals were housed in individual nonventilated cages in a temperature-controlled room (68 to 72°F) with a 12/12 hour light/dark cycle. Water and rat chow were provided *ad libitum*.

### Four-point bending used as a fatigue model

In order to induce microdamage in the tibiae of hindlimb-suspended and weight-bearing animals, two criteria needed to be fulfilled: (1) The model would have to be able to induce repeatable amounts of microdamage noninvasively within a moderate amount of time (1 to 2 hours), and (2) the model could not cause any alterations in animal behavior after loading (i.e., the animals must regain full usage of the hindlimbs shortly after loading).

The model chosen was based on the four-point bending model developed by Turner and colleagues.(31) In order to determine the initial effects of hindlimb suspension in combination with loading, two male 8-month-old Sprague-Dawley rats were hindlimb suspended for 14 days. At day 14, the rats were anesthetized, and the left tibiae were loaded for 7200 cycles at 2 Hz using a sinusoidal waveform with a peak load of 107.8 N (ΔLoad = 50.2 N), resulting in a maximum lateral strain of –7000 µstrain (see “In vivo strain gauge calibration for load parameters” below for further details). The 7000 µstrain level was chosen owing to the inability to achieve fatigue failure at a 4000 to 6000 µstrain level within 2 hours (done in another cohort). A 7000 µstrain level was found to achieve fatigue failure at approximately 1.5 to 2 hours. Hence the loading protocol was chosen to be 1 hour at this strain level. After loading, the animals were hindlimb suspended for an additional 3 days to observe any behavioral changes owing to loading and subsequently were euthanized. At sacrifice, the tibiae were dehydrated, stained with basic fuchsin, embedded in polymethyl methacrylate (PMMA), and sectioned at the region of interest (center at 8 mm proximal to tibia-fibula junction with a total length of 5 mm). The sections were examined using confocal microscopy.

Confocal microscopic examinations revealed that significant damage could be induced at the region of interest compared with the nonloaded control tibia (determined qualitatively). In addition, no abnormal behavior was observed after loading.

The high strain levels chosen (compared with 4000 µstrain) might induce more osteocyte apoptosis. However, since we are examining the effects of microdamage and thus, in effect, the response to osteocyte apoptosis, any additional apoptosis to potentially elevated strain levels was deemed acceptable. In addition, since the loading parameters would be similar between any experimental groups, any additional apoptosis should be similar in magnitude.

### In vivo strain-gauge calibration for load parameters

In order to determine the load parameters required to induce a strain level of –7000 µstrain at the lateral side of the mid-diaphysis of the tibia undergoing four-point bending, the strain–applied force relationship was determined for six 8-month-old male Sprague-Dawley rats that were split into two groups: (1) hindlimb suspension (HS) for 14 days (*n* = 3) and (2) normal weight bearing (WB) for 14 days (*n* = 3). At day 14, all rats were anesthetized, and a small incision was made at the lateral side at the mid-diaphysis of the tibiae. This allowed strain gauges to be placed bilaterally on the lateral side of the tibiae, 8 mm proximal to the tibia-fibula junction. For this, uniaxial EA-06-015DJ-120/LE strain gauges (Vishay Micro-Measurements, Raleigh, NC, USA) were trimmed to 4 × 1.5 mm and attached with Insta-Cure+ cyanoacrylate (Bob Smith Industries, Inc., Atascadero, CA, USA).

The average slope of the lateral strain versus applied force relationship was determined for each group [WB: –65.15µɛ/N (SD 6.31); HS: –56.55 µɛ (SD 18.87)]. No significant difference was found between the HS and WB groups, indicating that the loading regime induces similar strain magnitudes for weight-bearing and hindlimb-suspended animals at day 14. Based on the findings, a slope of –65.15µɛ/N was chosen, resulting in a lateral strain versus applied force relationship of


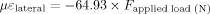


This relationship would be used for all the loading parameters in the subsequent experiment.

### Experimental protocol

After acclimation (day 0), 160 rats were assigned to one of three groups: weight bearing (WB, *n* = 60), hindlimb suspension (HS, *n* = 60), or hindlimb suspension with daily intermittent weight bearing following damage-inducing loading (HW, *n* = 40). Owing to the progressive development of the experimental hypothesis based on the WB and HS groups, the HW group was obtained and acclimated 4 months later than the WB and HS groups. Within each group, the rats were further divided into subgroups (*n* = 20) corresponding to three sacrifice time points [day 14 (WB and HS only), day 18, or day 35]. Animals assigned to the HS and HW groups were briefly anesthetized with an isoflurane (2%)–oxygen balance and hindlimb suspended using a custom-made hindlimb suspension system that is adaptable with standard SPF-rated ventilated no. 3 rat boxes. Unpublished work has successfully shown that the custom-made model induces similar physiologic changes as previous models,(16,18,32) where the disuse condition of the hindlimbs resulted in a decrease in bone formation and increase in bone resorption, whereas the general well-being of the hindlimb-suspended animals was maintained.(33)

At day 14, all animals were anesthetized, and their left tibiae underwent four-point bending to produce fatigue-induced microdamage. Specifically, the left tibia underwent a sinusoidal loading regime (7200 cycles at 2 Hz) with a maximum and minimum load of 107.8 and 57.6 N, respectively. This induced a maximum lateral strain of −7000 µstrain at the mid-diaphysis for both the HS and WB groups. Previous work had shown that loading the right tibia in a “nonbending” configuration of the four-point bending setup, as done by Turner and colleagues,(31) induced strain levels between −1000 to −400 µstrain at the lateral side for the prescribed sinusoidal loading regime. Turner and colleagues used this configuration of their four-point bender to evaluate the effects of just pinpoint loading to the bone and muscle tissue by loading the control leg in a nonbending fashion. For our setup, the prescribed nonbending loading regime was not sufficient to induce microdamage within the region of interest (ROI) 8 mm proximal to the tibia-fibula junction at the point of maximum bending. However, it was found that at the points of contact between the loading pads of the four-point bending setup, significant amounts of microdamage were induced. To test the hypothesis adequately, it thus was determined that any microdamage induced at the application of load for the control load would result in remodeling, which would skew the findings owing to remodeling of the ROI of the left tibia undergoing pure bending. Therefore, the right tibia would serve as a nonloaded, completely undamaged control.

Once the loading regime was complete, animals were allowed full recovery from anesthesia and subsequently hindlimb suspended or allowed full weight bearing again, in correspondence with their group assignment. Starting at day 15, animals in the HW group were unhooked from the tail-suspension mechanism in their cages and allowed 1 hour of full weight bearing within their respective cages each day, after which they were returned to hindlimb suspension.

At sacrifice, right-left pairs of tibiae were carefully dissected free of soft tissue and assigned to one of three treatments within each subgroup: flow cytometry for hematopoietic stem cell (HSC) and monocyte markers (*n* = 6), basic fuchsin staining for microdamage assessment (*n* = 7), or histologic and immunohistochemical staining (*n* = 7). Prior to staining, morphologic analysis was conducted on the last two groups using 3D micro-computed tomography (µCT).

### Micro-computed tomography (µCT)

Once carefully dissected free of soft tissue, the tibiae not used for flow cytometry were scanned on a µCT system (GE Healthcare Systems, London, ON, Canada) and reconstructed with a voxel size of 25 µm. Morphologic parameters were determined for the tibiae at the cortical ROI that experienced the maximum bending during four-point bending (i.e., at the mid-diaphysis). Specifically, the ROI had a length of 4 mm, with its center located 8 mm proximal to the tibia-fibula junction. Bone architectural parameters for this ROI were determined using a custom analysis program and a commercially available voxel analysis software package (MicroView, Version 2.2, GE Healthcare). The following parameters were calculated: tissue mineral content (TMC), tissue mineral density (TMD), cross-sectional moment of inertia (*I*_*zz*_), and cortical and marrow area.

### Flow cytometry (FACS)

Specimens assigned for flow cytometry were placed in PBS + 2% normal calf serum (NCS) on ice immediately after dissection. Marrow was flushed from the tibiae and washed in PBS + 2% NCS, and 10^6^ cells were removed and put on ice for subsequent staining. To reduce background noise from red blood cells in the flushed marrow, the collected cells were resuspended briefly in Ack lysis buffer and subsequently washed in PBS + 2% NCS. To prevent nonspecific binding of selected antibodies, cells were incubated with rIgG, mIgG, and rIgAκ (BD Biosciences Pharmingen, San Jose, CA, USA) for 15 minutes at 4°C prior to staining. In order to determine the effect of the experimental conditions on the osteoclast lineage, the cells were incubated for 25 minutes at 4°C with the following antibodies: anti-mouse CD 117 (c-kit), hematopoietic stem cell (HSC) marker, PE-conjugated, isotype: rat IgG2bκ, clone: 2B8 (Beckman Coulter, Inc., Fullerton, CA, USA); anti-rat CD11b (Mac-1 α chain), monocyte-macrophage marker, FITC-conjugated, isotype: mouse IgAκ, clone: WT.5 (BD Biosciences Pharmingen).

Previous work(34) has shown that the 2B8 clone for mouse CD117 cross-reacted with rat CD117 by FACS (Santa Cruz Biotech, Santa Cruz, CA, USA). In addition, positive staining was determined with the selected CD117 antibody by achieving similar staining for control rat tibiae marrow cells and C57 mouse tibiae marrow cells (data not shown).

Once stained, cells were analyzed using a FACS Calibur (BD Biosciences). For each sample, 30,000 events were collected. For analysis of the flow cytometric data, the forward scatter and side scatter gate was set to a region that has been shown previously to include stem cells and monocytes.(35) The CD117+ and CD11b+ populations within the gate were identified as cells expressing specific levels of fluorescent activity above the nonspecific staining and autofluorescence of the isotype control.

Owing to insufficient cell counts during FACS, 7 animals had to be eliminated from the flow cytometric data, resulting in final groups at day 14: WB (*n* = 6)/HS (*n* = 5); day 18: WB (*n* = 3)/HS (*n* = 4)/HW (*n* = 7); and day 35: WB (*n* = 4)/HS (*n* = 6)/HW (*n* = 7).

### Basic fuchsin staining

On dissection and prior to µCT scanning, specimens assigned for basic fuchsin staining were kept in 70% ethanol. After µCT scanning, the tibiae were completely dehydrated and stained with basic fuchsin according to the protocol of Burr and colleagues.(36) Subsequently, they were embedded in Koldmount fast-curing cold monomer (IDP/Vernon-Benshoff Co., Albany, NY, USA) and sectioned 400 µm transverse to the longitudinal axis of the tibia at the µCT ROI using a Buehler Isomet low-speed diamond-blade saw. The section closest to the center of the ROI for each tibia was mounted on a plastic microscope slide using cyanoacrylate and polished to a final thickness of 150 to 200 µm. The sections were examined with a standard confocal microscope (Zeiss LSM 510-META Laser Scanning Confocal Microscope, Carl Zeiss MicroImaging, Inc., Thornwood, NY, USA) at 40 × magnification using an HeNe1 laser (543 nm) with a Texas red/rhodamine filter. Images were taken for the entire cortical region and subsequently analyzed using the Zeiss LSM Image Browser (Version 4.2.0.121, Carl Zeiss Micro Imaging, Inc.) to quantify linear microdamage within the cortical region (see 4*A*)

**Fig. 4:**
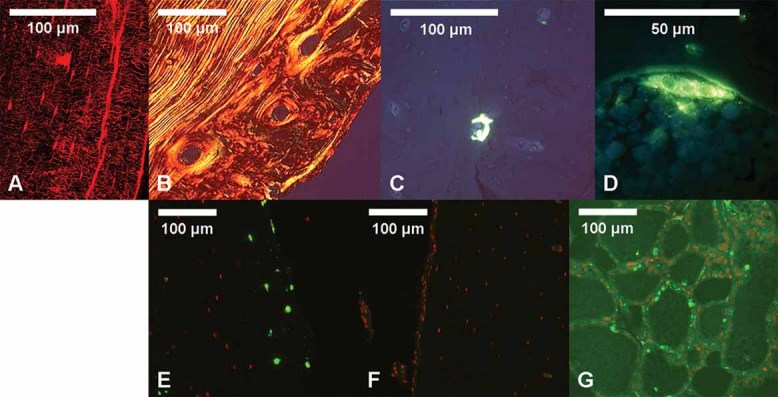
Representative histologic images. (*A*) Confocal image of basic fuchsin–stained microdamage. (*B*) Polarized light microscopic image of picro–sirius red–stained woven and cortical bone. (*C*) Fluorescent microscopic image of ELF97 TRAP+ resorption pit. (*D*) Close-up fluorescent microscope image of ELF97 TRAP+ osteoclast. (*E*) Fluorescent microscope image of Apoptag-stained cortical bone with induced microdamage (from left-loaded tibia). Apoptotic osteocytes exhibit bright green fluorescence (*wam*). (*F*) Fluorescent microscope image of Apoptag-stained cortical bone without induced microdamage (from nonloaded contralateral right tibia). (*G*) Fluorescent microscope image of Apoptag-stained female rodent mammary glands serving as positive controls.

Only linear microdamage was quantified because it has been shown that remodeling occurs only in bone with linear microcracks, whereas bone containing only diffuse damage fails to initiate a remodeling response.(37)

Using light microscopy, the cortical cross-sectional areas were calculated with Bioquant image software (BQ OSTEO, Version 7.20.10, Bioquant Image Analysis Corporation, Nashville, TN, USA) while omitting woven bone areas.

### Histology and immunohistochemistry

On dissection and prior to µCT scanning, specimens assigned for histology and immunohistochemistry were immediately placed in 10% neutral buffered formalin (NBF) for 2 days, followed by 70% ethanol. Subsequent to µCT scanning, specimens were decalcified over 5 weeks using 10% EDTA at 4°C, after which specimens were paraffin embedded. The bone within the µCT ROI was sectioned 7 µm transverse to the longitudinal axis of the tibia.

#### ELF97 (TRAP)

Two paraffin-embedded sections per tibia (1 mm apart within the µCT ROI, with the first section 7 mm proximal to the tibia-fibula junction) were stained with ELF97 phosphate (Molecular Probes, Eugene, OR, USA) to visualize TRAP+ resorption pits within the cortical and woven bone. The protocol used for the fluorescence-based ELF97 TRAP stain was adapted from the in vitro ELF97 stain protocol developed by Filgueira.(38) Specifically, 50 µL of ELF97 reaction mix (41.15 µL dH_2_O, 0.55 µL sodium nitrite, 5.00 µL 2 mM ELF97, 2.20 µL acetate, and 1.1 µL tartrate) was applied to each selected section, which was incubated at room temperature in the dark for 5 minutes. Sections were rinsed subsequently in dH_2_O, and cover slips were applied using Prolong Gold antifade reagent (Invitrogen/Molecular Probes). Sections were imaged immediately using appropriate fluorescent filters (see 4*C,D*). Histologic measurements included TRAP+ intracortical resorption pits per cortical area, percent TRAP+ endosteal perimeter (%TRAPendo), and percent TRAP+ periosteal perimeter (%TRAPperi). These measurements were determined while omitting woven bone areas. The average measurements for the two sections were used for the subsequent analysis.

#### Picro-sirius red

Two sections per tibia (1 mm apart within the µCT ROI, with the first section 7 mm proximal to the tibia-fibula junction) were stained with picro-sirius red F3BA in order to quantify woven bone apposition.(39,40) Using polarized light microscopy, collagen fibers were highlighted to distinguish the lamellar and woven bone and enable quantification of woven bone apposition at the periosteal surfaces using Bioquant image software (BQ OSTEO Version 7.20.10) (see 4*B*). Histologic measurements were done of woven bone area. The average measurements for the two sections were used for the subsequent analysis.

#### Immunohistochemical apoptosis detection (Apoptag)

One section per tibia (taken at the center of the µCT ROI) was used for immunohistochemical osteocyte apoptosis detection using the ApopTag Plus Fluorescein In Situ Apoptosis Detection Kit (Chemicon/Millipore, Temecula, CA, USA). Specifically, the assay detects apoptosis via fluorescent DNA fragmentation labeling, similar to a standard TUNEL assay.(41) Once stained, sections were cover slipped using propidium iodide–antifade solution (Millipore) (see 4*E, F*). Sections of female rodent mammary glands were used as positive controls (see 4*G*) owing to extensive apoptosis occurring in the tissue 3 to 5 days after weaning of rat pups.(42) All sections were imaged immediately using appropriate fluorescent filters. Histologic measurements included number of apoptosis-positive osteocytes per cortical area. These measurements were determined while omitting woven bone areas.

### Statistics and graph nomenclature

**Fig. 1:**
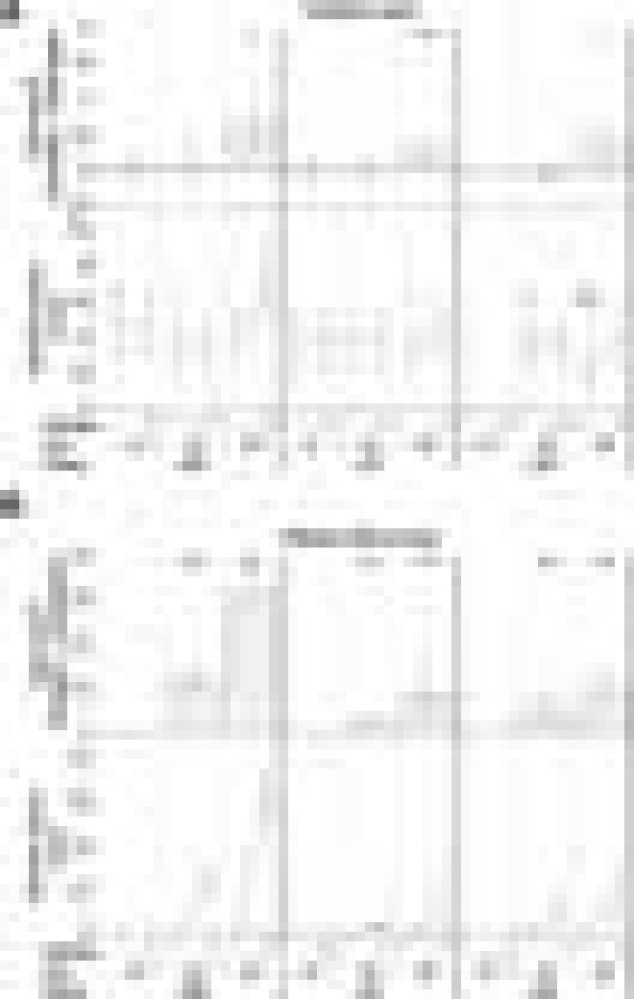
Cross-sectional parameters for undamaged (–) and damaged (+) tibiae. (*A*) Cortical area including woven bone determined from µCT. Animals per group (WB/HS/HW): Day 14 (13/14/–), day 18 (14/15/13), day 35 (14/17/13). (*B*) Woven bone area. Animals per group (WB/HS/HW): day 14 (6/7/–), day 18 (7/7/6), day 35 (7/8/6). Statistics: (*a*) Indicates significant difference between right versus left leg for that group on the particular day (i.e., Δ is significantly different from zero). (*b*) Indicates significant difference between WB and group on that day. (*c*) Indicates significant difference between HS and HW on that day. (*e*) Indicates significant difference between days 18 and 35 within the specific group (WB, HS, or HW). (*f*) Indicates significant difference between days 14 and 18 within the specific group (WB, HS, or HW). Error bars indicate standard deviations.

Results are presented graphically both in absolute values for each individual leg and as the difference between the damaged (+) and undamaged (–) contralateral legs (Figs. 1 through 3). The term *delta* (Δ) is used to indicate the differences between contralateral limbs (damaged tibia – undamaged tibia).

**Fig. 2:**
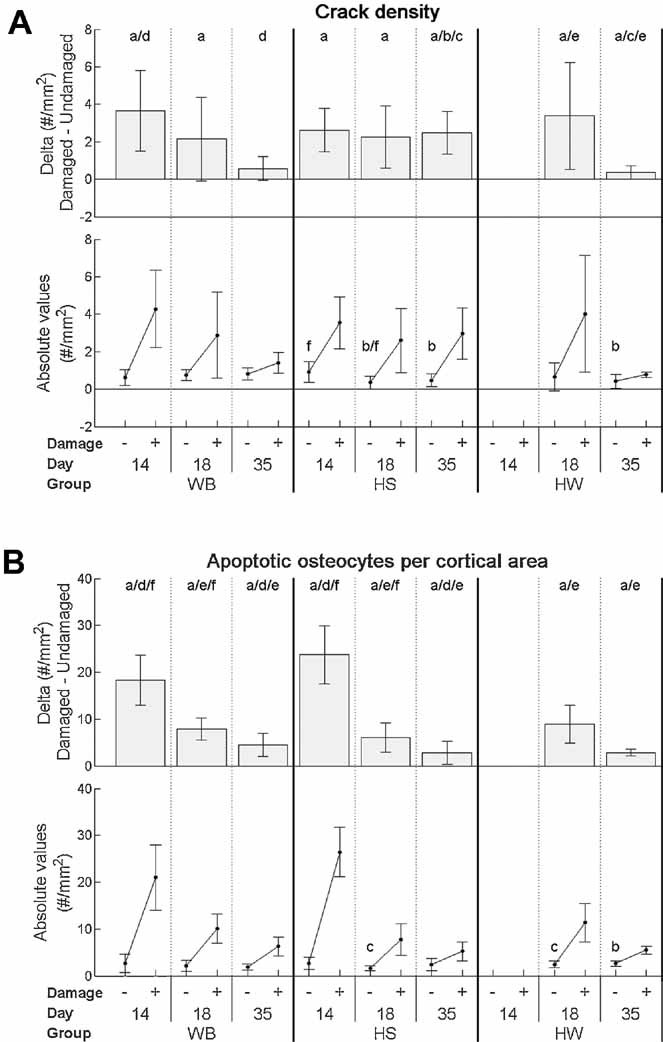
Microdamage parameters and associated osteocyte apoptosis for undamaged (–) and damaged (+) tibiae. (*A*) Crack density. Crack density was determined while omitting woven bone areas. Animals per group (WB/HS/HW): Day 14 (7/6/–), day 18 (7/8/7), day 35 (7/9/7). (*B*) Apoptotic osteocytes per cortical area. Number of apoptotic osteocytes (per cortical area) was determined while omitting woven bone areas. Animals per group (WB/HS/HW): Day 14 (6/7/–), day 18 (7/7/6), day 35 (7/8/6). Statistics: (*a*) Indicates significant difference between right versus left leg for that group on the particular day (i.e., Δ is significantly different from zero). (*b*) Indicates significant difference between WB and group on that day. (*c*) Indicates significant difference between HS and HW on that day. (*d*) Indicates significant difference between day 14 and day 35 within the specific group (WB, HS, or HW). (*e*) Indicates significant difference between days 18 and 35 within the specific group (WB, HS, or HW). (*f*) Indicates significant difference between days 14 and 18 within the specific group (WB, HS, or HW). Error bars indicate standard deviations.

**Fig. 3:**
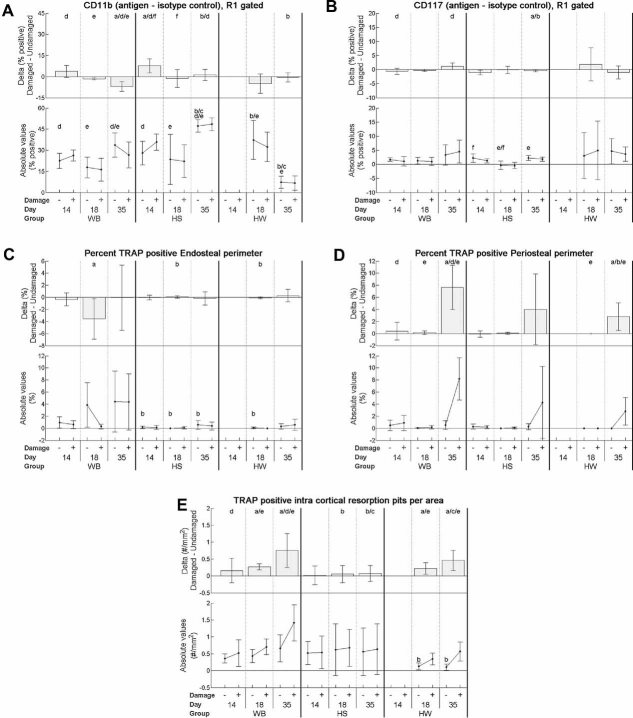
Monocyte and HSC flow cytometry markers for tibial bone marrow and TRAP-stained parameters for undamaged (–) and damaged (+) tibiae. (*A*) CD11b, monocyte marker. (*B*) CD117, HSC marker. Animals per group for both CD11b/CD117 (WB/HS/HW): Day 14 (6/5/–), day 18 (3/4/7), day 35 (4/6/7). (*C*) Percent TRAP+ endosteal perimeter. (*D*) Percent TRAP+ periosteal perimeter. (*E*) TRAP+ resorption pits. Measurements were determined while omitting woven bone areas. Animals used per group for TRAP parameters (WB/HS/HW): Day 14 (6/7/–), day 18 (7/7/6), and day 35 (7/8/6). Statistics: (*a*) Indicates significant difference between right versus left leg for that group on the particular day (i.e., Δ is significantly different from zero). (*b*) Indicates significant difference between WB and group on that day. (*c*) Indicates significant difference between HS and HW on that day. (*d*) Indicates significant difference between day 14 and day 35 within the specific group (WB, HS, or HW). (*e*) Indicates significant difference between days 18 and 35 within the specific group (WB, HS, or HW). (*f*) Indicates significant difference between days 14 and 18 within the specific group (WB, HS, or HW). Error bars indicate standard deviations.

Statistical analyses were done for the right undamaged tibiae (serving as internal systemic controls) and delta measurements. To compare damaged with undamaged contralateral sides, paired *t* tests were used. A two-way ANOVA with a post hoc correction was used between experimental groups (WB/HS/HW) and between groups at different time points for both absolute right tibiae values and delta values. Significance was defined as *p* ≤ .05. The analysis was performed using SPSS statistical software (SPSS, Inc., Chicago, IL, USA).

In all figures (for both absolute values and delta), significance is denoted with a letter in the range *a* to *f*: (*a*) indicate significant difference between right versus left leg for that group on the particular day; (*b*) significant difference between WB and group on that day; (*c*) significant difference between HS and HW on that day, (*d*) significant difference between day 14 and day 35 within the specific group (WB, HS, or HW), (*e*) significant difference between days 18 and 35 within the specific group (WB, HS, or HW), and (*f*) significant difference between days 14 and 18 within the specific group (WB, HS or HW). Error bars in all figures indicate standard deviations.

## Results

During the experimental protocol, seven animals had to be euthanized and replaced owing to complete fracture during 4-point bending (WB: 1; HS: 3) or to failure in health maintenance indicated by weight loss of more than 15 percent of initial body weight (HS: 2, HW: 1).

Significant initial woven bone formation occurred for all three groups. Over time, mineralization increased for all groups, but with the WB group having significantly more woven bone deposited than the HS and HW groups (1*B*), leading to a larger cross-sectional area (1*A*). The woven-bone response resembled the pattern of a stress-fracture response. In addition, µCT showed that tissue mineral content (TMC), tissue mineral density (TMD), and cross-sectional moment of inertia (*I*_*zz*_) followed similar significant trends as cortical area across groups and time (not shown). Both the HS and HW groups showed a significant increase in delta marrow area at day 35.

Quantified confocal microscopic measures of basic fuchsin–stained sections revealed that the resulting microdamage (see 4*A*) looked similar in nature to microdamage induced in the rat ulnae with lower loads for longer loading durations.(5,43–45) In addition, it was shown that significant amounts of microdamage were equally induced at the time of microdamage creation (day 14) for the WB and HS groups (2*A*). The WB and HW groups showed a significant decrease in microdamage over time, indicating that microdamage resorption had occurred. In contrast, significant damage remained in the HS group for the same time period (see 2*A*). Interestingly, the significant decrease in crack density (Cr.Dn.) for the HW group occurred from day 18 to day 35, whereas this occurred between day 14 and day 35 for the WB group. This could indicate a delayed resorption response for the HW group compared with the WB group.

Detection of osteocyte apoptosis revealed that the damage induced by fatigue loading resulted in similar and significant increases in apoptotic osteocytes in the cortical bone for all three groups at all time points (see 2*B*). The number of apoptotic osteocytes decreased significantly for all groups over time but remained similar between all groups throughout (see 2*B*).

FACS was used to investigate the osteoclast lineage of the bone marrow. An early marker (CD117) was used for hematopoietic stem cells, whereas the intermediate marker (CD11b) was used for evaluation of the monocyte population. The results showed a systemic increase in CD11b from days 14 to 35 for both the WB and HS groups, whereas a decrease was found for the HW group (3*A*). However, ΔCD11b was significantly decreased across time for the WB group, indicating that the damaged leg had fewer circulating monocytes relative to the contralateral control limb. The change in ΔCD 11b for the WB group was significantly different from that in both the HS and HW groups at day 35.

The results for CD117 showed no differences in systemic values between groups (see 3*B*), whereas it was found that the HS group had a significant systemic reduction in CD117 at day 18 relative to both days 14 and 35. A significant increase in ΔCD117 across time was found for the WB group, resulting in a significant difference in ΔCD117 compared with the HS group at day 35 (see 3*B*).

TRAP staining using ELF97 phosphate showed that control levels of %TRAPendo were significantly lower for the HS group than for the WB group at all time points (see 3*C*). It was found that delta %TRAPendo was significantly different between the WB group and both the HS and HW groups at day 18 (see 3*C*). In addition, the WB and HW groups had a significant increase in %TRAPperi at day 35, with the increase for the WB group being significantly greater than for the HW group (see 3*D*).

Histology for TRAP also demonstrated a significant increase in ΔTRAP+ intracortical resorption pits following loading for the WB and HW groups, which was completely absent for the HS group (see 3*E*). In addition, the HW and HS groups were significantly different at day 35 (see 3*E*). Finally, it was found that the control level of TRAP+ intracortical resorption pits of the right, undamaged tibiae for the HW group were significantly lower than for the WB group at days 18 and 35.

## Discussion

The damage response corresponds to what has been shown in previous animal models, where fatigue loading induced woven bone formation that was both dependent on and proportional to the amount of induced microdamage.(43,44,46) Periosteal woven bone formation after fatigue damage also has been shown to aid in the rapid recovery of whole-bone strength while increasing fatigue life.(44,47) Hence not only does a significant amount of damage remain in animals that were hindlimb suspended, but the protective mechanism of woven bone formation was not present, suggesting that whole-bone strength remains low in a disuse setting following fatigue damage even with daily short-term physiologic loading. One explanation for the significantly reduced woven bone response for the HS and HW groups may be a significant reduction in blood flow. Studies using HS models have demonstrated reduced blood flow,(22) and it has been shown that there is a correlation between increased fatigue loading, increased vascularity, and increased woven bone formation.(48) However, it also has been shown that 1 hour of daily loading for hindlimb-suspended animals appears to prevent adverse changes in myocardial contractility and therefore blood flow.(28) Hence the significantly smaller woven bone response for the HW and HS groups seems most likely to be stemming from the reduction in osteoblast responsiveness and bone-formation rate associated with hindlimb suspension.(19,49)

Previous studies have shown that excessive fatigue loading results in woven bone formation in addition to increased intracortical resorption. The intracortical resorption response, which has been observed separately in several studies,(2,4–8) was evident for both the WB and HW groups following fatigue damage but not for the HS group. The absence of the intracortical resorption response could have been due to changes in the initial osteocyte response to the induced microdamage. However, the evidence from immunohistochemistry indicates that this response was not altered by disuse. In addition, a similar decay of apoptotic osteocytes over time (owing to assay technique) was observed for all groups, indicating that although microcrack removal was rescued for the HW group, it was not due to an increase in initial cell apoptosis, which could have changed the amount of osteoclast recruitment. Hence similar amounts of induced microdamage resulted in similar amounts of osteocyte apoptosis for all groups.

The lack of microdamage removal and absence of intracortical resorption pits for the HS group could be due to either a loss of the “targeting” mechanism of remodeling or a lack of osteoclast recruitment following microdamage. In addition, the potential delay in microdamage removal for the HW group relative to the WB group could be due to the much shorter daily loading duration of the HW group or the physiologic assimilation to daily hourly loading following 14 days of constant hindlimb suspension.

Examining the HSC and monocyte population of the osteoclast lineage with flow cytometry revealed a significant increase in systemic monocytes for both the WB and HS groups potentially owing to the overall effect of the direct periosteal trauma. However, over time, the damaged leg showed a significant reduction in monocytes relative to the control leg for the WB group. This suggests a potential shift in the osteoclast lineage, with a larger amount of monocytes being differentiated in the damaged leg for the WB group owing to the recruitment of osteoclasts to the damaged regions. This “depletion” of monocytes would trigger a demand for more, resulting in an increase in the HSC population for the WB group, as suggested by the increase in ΔCD117 over time. Hence the flow cytometry results indicate that the histologic evidence of “no damage removal” at day 35 for HS was due to a lack of osteoclast activation and not a change in the targeting mechanisms of remodeling. Although the HW group showed increased microdamage removal over time, the initial increase in systemic monocytes relative to the WB group was followed by a significant decrease, which could be due to a complete shift in the osteoclast lineage.

TRAP staining indicated that intracortical resorption following microdamage is deactivated during disuse and that physiologic loading is necessary for the remodeling repair response that follows significant accumulation of microdamage. The decrease in basal level of TRAP+ intracortical resorption pits of the right, undamaged tibiae for the HW group (compared with the WB and HS groups) could be due to the later point in time that the group was acquired. This is supported by studies showing seasonal changes occurring in bone despite a constant diet, established light/dark cycles, and a steady temperature.(51,52) Despite the possibility of these seasonal changes, TRAP staining for the HW group reaffirmed that targeted resorption was rescued owing to intermittent physiologic loading during disuse.

The lack of osteoclast activation for the HS group could be due to a decrease in interstitial fluid flow that results from disuse,(23) particularly given that several studies suggest that load-induced fluid flow may be necessary to provide sufficient transport of larger molecules such as proteins to and from osteocytes.(24,25) In addition, the evidence that osteocyte apoptotic bodies induce osteoclastogenesis leading to localized bone resorption(53) suggests that during hindlimb suspension or disuse, the “activating” signal for resorption of microdamage may be related to a lack of fluid flow through the canalicular system, resulting in inhibited delivery of resorption-initiating signals from the apoptotic osteocytes. Furthermore, the results for the HW group may indicate that intermittent loading during disuse can provide enough interstitial fluid flow to distribute any “active” signal of resorption.

These findings are supported by recent studies by Tatsumi and colleagues(54) and Cardoso and colleagues,(9) who both demonstrated a causal relationship between osteocyte apoptosis and activation of bone resorption. While Cadoso and colleagues(9) demonstrated this with fatigue-induced osteocyte apoptosis, the study by Tatsumi and colleagues(54) used a transgenic mouse model for inducible and specific osteocyte ablation through apoptosis. The work by Tatsumi and colleagues parallels our findings by showing a trend toward a decrease in trabecular osteoclasts in addition to a significant reduction in RANKL expression for tail-suspended versus ground-based osteocyte-ablated transgenic mice. Similar to our findings, this would indicate that the removal of physiologic loading leads to a lack of osteoclast activation.

There are a number of limitations associated with this study. The decrease in apoptotic osteocytes over time may be associated with the method of apoptosis detection. The ApopTag Plus Fluorescein In Situ Apoptosis Detection Kit detects apoptosis via fluorescent DNA fragmentation labeling, similar to a standard TUNEL assay. The kit can detect early-stage apoptosis but also will stain DNA fragments in apoptotic bodies. Since the apoptotic cycle is completed in 1 to 2 days, the early apoptotic osteocytes are now replaced with nonattached apoptotic bodies within the lacunae. The processing of the tissue from days 18 and 35 could remove these bodies, leaving an empty lacuna. For this study, only early apoptotic osteocytes were accounted for, thus revealing a decrease from day 14 to day 35, similar to that observed from day 3 to day 7 for vehicle-treated rats with induced bone microdamage.(55) In addition, the focus of the apoptosis assay was to determine if the amount of apoptotic osteocytes was similar between groups immediately following microdamage because different osteocyte apoptotic baselines between groups could have given rise to a different osteoclast response.

A second unexpected observation is the absence of an increase in apoptotic osteocytes for the right, nondamaged tibia across time for all groups (see 2*B*). This is countered by the work by Aquirre and colleagues,(56) who demonstrated an increase in apoptotic vertebral cortical osteocytes for hindlimb-suspended adult mice. The reasons for this discrepancy could be several: (1) the difference in the method of osteocyte apoptosis detection and (2) the rate or occurrence of osteocyte apoptosis owing to hindlimb suspension could be different between the vertebral body and the tibia. Basso and colleagues(57) showed a significant increase in apoptotic osteocytes in the tibia of male rats with 2 weeks of hindlimb suspension. This was done in 5-week-old animals, however, which might exhibit a different rate of apoptosis than a more skeletally mature animal. Another explanation for the observation of no changes (or perhaps delayed changes) in osteocyte apoptosis could be the ability of our rats to maintain weight beyond an initial assimilation period (Tables 3 and 4). Tables 1 through 3 contain previously unpublished data from our hindlimb-suspension model development that demonstrated the effects of pure hindlimb suspension and weight bearing up to 35 days. As seen in Table 1, 35 days of hindlimb suspension did not decrease the cortical area significantly (although significant cancellous bone was lost). This parallels the studies by Bloomfield and colleagues,(19) who showed an increase or maintenance in cortical area with a decrease in trabecular parameters when mature rats maintained their weight over 4 weeks of hindlimb suspension. In contrast, studies by Hefferan and colleagues,(58) Allen and colleagues,(59) and Vico and colleagues(60) showed early decreases in cortical area and trabecular bone volume fraction (BVF) during 2 to 4 weeks of HS in mature rats who lost significant weight continuously during the hindlimb-suspension period. Hence longer periods of hindlimb suspension might result in significant increases in apoptotic osteocytes in our model[Table tbl2].

**Table 1:** Data for Hindlimb Suspension Animal Model Development: Table with Tibial µCT Data for Pure Control and HS (No Microdamage Induction)

		Control (WB)	Hindlimb suspension (HS)	
			
	Day	35	14	35
	*n*	5	2	14
Cortical area (mm^2^)	Avg.	6.36	6.68	6.30
	SD	0.45	0.28	0.61
Trabecular BVF (BV/TV)	Avg.	0.37	0.37	*0.28*
	SD	0.03	0.05	*0.06*

Italic = *p* < .01 versus control (WB) day 35. ROI parameters: Length of 5 mm with its center located 8 mm proximal to the tibia-fibula junction.

**Table 2 tbl2:** Data for Hindlimb Suspension Animal Model Development: Table with Serum Data for Pure Control and HS (No Microdamage Induction)

		Control (WB)			Hindlimb suspension (HS)		
			
	Day	0	21	35	0	21	35
	*n*	5	5	5	10	9	8
Osteocalcin, ng/mL	Avg.	2.47	1.72	2.07	1.93	1.39	1.32*
	SD	1.93	0.78	0.52	0.83	0.85	0.48
TRAP5b, U/L	Avg.	0.77	0.94	0.88	0.85	1.16**	1.16**
	SD	0.19	0.20	0.11	0.17	0.43	0.30

Serum TRACP5b (RatTRAP, SBA Sciences) and OCN (Rat Osteocalcin, BT-490, Biomedical Technologies) were measured by ELISA [absorbance measured at 450 nm (OCN) and 405 nm (TRACP5b)] in order to determine relative systemic expression of markers of bone resorption and formation, respectively.

* *p* = .07 versus HS day 0.

** *p* = .07 WB versus HS for that day.

**Table 3:** Table with Body Mass Data for Pure Control and HS (No Microdamage Induction)

		Control (WB)		Hindlimb suspension (HS)			
			
	Day	21	35	14	21	28	35
	*n*	5	10	4	9	6	14
% Body mass change compared to day 0	Avg.	*8.30*	*9.36*	*−6.74*	*−5.28*	*−4.12*	*−5.75*
	SD	1.90	3.43	3.29	5.91	5.05	5.99

Italic = value significantly different from zero (*p* < .05).

**Table 4:** Table with Body Mass Data for WB, HS, and HS (with Microdamage Induction)

		WB			HS			HW		
				
	Day	14	18	35	14	18	35	14	18	35
	*n*	60	20	20	66	20	23	40	20	20
% Body mass change compared with day 0	Avg.	*2.7*	*1.8*	*8.4*	*−4.2*	*−6.7*	*−4.4*	*−2.9*	*−5.5*	*−3.4*
	St.Dev.	2.8	3.2	2.8	4.1	5.9	4.4	3.8	4.4	4.5

*Italic* = value significantly different from zero (*p* < .05).

A final limitation relates to the direct periosteal trauma and reaction caused by the loading pads of the four-point loading system to the cortical contact region. Owing to the direct loading of the periosteum, the sections for histology all were taken at the center of the ROI, that is, approximately 5.5 mm away from the nearest loading point. By doing so, the effect of direct loading was minimized or absent owing to the relatively great distance away from the loading points. However, the potential effect of direct periosteal trauma on the marrow constituents used for flow cytometry cannot be discerned from the effect owing to microdamage.

Despite the limitations, the data suggest that individuals with severely limited activity potentially may accumulate unrepaired microdamage, thereby increasing fracture risk. The change in remodeling owing to disuse also may influence how stress fractures are treated. The current treatment of stress fractures by casting and/or prevention of load bearing may need to be reconsidered because the repair of microdamage may proceed more effectively when combined with physiologic loading. Early evidence to support this hypothesis has been found in treatment of running injuries. Standard treatment methods for stress fractures associated with long-distance running typically prescribe up to 12 weeks of therapy (dominantly non–weight bearing) before returning to a normal running schedule.(29) Recent studies, however (without an experimental basis), have decreased the recovery period by implementing earlier cross-training, enabling the athlete to return to function in only 7 weeks.(30) The results from these early clinical studies parallel the results in this study by demonstrating that an intermittent controlled therapeutic loading regime of the injured limb potentially could increase the rate and extent of recovery from a stress fracture owing to the increase in targeted remodeling.

In summary, this study demonstrates that physiologic loading is necessary for a remodeling repair response to occur following significant accumulation of microdamage. In addition, intermittent daily physiologic loading can reverse the loss of remodeling in response to microdamage during disuse. Although intermittent loading cannot rescue the reduction in woven bone production following microdamage, the targeted resorption of microdamage is revived. Last and most important, while a number of studies have proposed that the repair of microdamage is triggered by cell apoptosis, these results demonstrate that this mechanism may be insufficient without the stimulation provided through physiologic loading.
